# The Physics of Smell

**Published:** 1898-12

**Authors:** 


### THE PHYSICS OF SMELL.

Had the sense of smell occupied in man the important position it bears in the hierarchy of senses of animals, it would no doubt have been the subject of a far greater amount of investigation. Sight and hearing have been subject to a long series of ingenious investigations, which have been the means of revealing their essential nature, but taste and smell have been comparatively neglected.

For this reason the address of Professor Ayrton on the physics of smell, before the British Association for the Advancement of Science, at Bristol, becomes especially interesting.

The sense of smell in man is developed so rudimentarily that it affords little opportunity for study. Our memory for odor is very imperfect, and our olfactory sensation possesses none of the definiteness and precision of other senses. It resembles so much that of taste that these two seem to have been deemed a common sensation.

In animals, of course, all this is very different, and they have gained, or we have lost, in definiteness of perception, so that any study of smell must be made elsewhere than in the human family.

Sexual odors are matters that should possess more than passing interest for the psychologist. Scarpa long ago showed that if he plunged his hand into water after handling a female toad, the males were attracted to it. Hounds will recognize by smell the trace of animals perfectly imperceptible to sight. Water, which has no smell to man, can be perceived by many animals at a great distance.

The diffusion of odors, especially those having a sexual bearing, would seem to be prodigious. Professor Ayrton found that if females of certain moths be exposed in a muslin bag, the males will soon be seen to congregate around them.

Professor Ayrton specially directed his attention to the smell of metals, the rapidity with which odors diffuse, and the capability of odors to traverse films of glass. In some important respects his inquiries may be said to have augmented our knowledge of the action of the special senses.—*Medical Age.*

